# Physical activity and risk of Amyotrophic Lateral Sclerosis in a prospective cohort study

**DOI:** 10.1007/s10654-016-0119-9

**Published:** 2016-03-11

**Authors:** Valentina Gallo, Nicola Vanacore, H. Bas Bueno-de-Mesquita, Roel Vermeulen, Carol Brayne, Neil Pearce, Petra A. Wark, Heather A. Ward, Pietro Ferrari, Mazda Jenab, Peter M. Andersen, Patrik Wennberg, Nicholas Wareham, Verena Katzke, Rudolf Kaaks, Elisabete Weiderpass, Petra H. Peeters, Amalia Mattiello, Valeria Pala, Aurelio Barricante, Maria-Dolores Chirlaque, Noémie Travier, Ruth C. Travis, Maria-Jose Sanchez, Hélène Pessah-Rasmussen, Jesper Petersson, Anne Tjønneland, Rosario Tumino, Jose Ramon Quiros, Antonia Trichopoulou, Andreas Kyrozis, Despoina Oikonomidou, Giovanna Masala, Carlotta Sacerdote, Larraitz Arriola, Heiner Boeing, Matthaeus Vigl, Francoise Claver-Chapelon, Lefkos Middleton, Elio Riboli, Paolo Vineis

**Affiliations:** Department of Epidemiology and Biostatistics, School of Public Health, Imperial College London, St. Mary’s Campus – Norfolk Place, W2 1PG London, UK; Barts and the London School of Medicine, Centre for Primary Care and Public Health, Queen Mary, University of London, London, UK; Italian National Institute of Health, Rome, Italy; National Institute for Public Health and the Environment, Bilthoven, The Netherlands; Department of Gastroenterology and Hepatology, University Medical Centre, Utrecht, The Netherlands; Division of Environmental Epidemiology, Institute for Risk Assessment Sciences (IRAS), Utrecht University, Utrecht, The Netherlands; Department of Public Health and Primary Care, University of Cambridge, Cambridge, UK; Epidemiology and Population Health, London School of Hygiene and Tropical Medicine, London, UK; International Agency for Research on Cancer (IARC), Lyon, France; Department of Pharmacology and Clinical Neuroscience, Umeå University, Umeå, Sweden; Department of Public Health and Clinical Medicine, Umeå University, Umeå, Sweden; Division of Cancer Epidemiology, German Cancer Research Centre (DKFZ), Heidelberg, Germany; Department of Community Medicine, Faculty of Health Sciences, The Artic University of Norway, Tromsö, Norway; Cancer Registry of Norway, Institute of Population-Based Cancer Research, Oslo, Norway; Department of Medical Epidemiology and Biostatistics, Karolinska Institutet, Stockholm, Sweden; Folkhälsan Research Center, University of Helsinki, Helsinki, Finland; Department of Epidemiology, Julius Center for Health Sciences and Primary Care, University Medical Center Utrecht, Utrecht, The Netherlands; Department of Clinical and Experimental Medicine, Federico II University, Naples, Italy; Epidemiology and Prevention Unit, Fondazione IRCCS Istituto Nazionale dei Tumori, Milan, Italy; Navarre Public Health Institute, Pamplona, Spain; Consortium for Biomedical Research in Epidemiology and Public Health (CIBER Epidemiología y Salud Pública-CIBERESP), Madrid, Spain; Epidemiology Department, Murcia Regional Health Council, Murcia, Spain; Unit of Nutrition, Environment and Cancer, Catalan Institute of Oncology (ICO), L’Hospitalet de Llobregat, Barcelona, Spain; Cancer Epidemiological Unit, Nuffield Department of Clinical Medicine, University of Oxford, Oxford, UK; Andalusian School of Public Health, Granada, Spain; Department of Neurology, Skåne University Hospital, Lund University, Malmö, Sweden; Danish Cancer Society Research Center, Copenhagen, Denmark; Ragusa Cancer Registry, Azienda Ospedaliera “Civile MP Arezzo”, Ragusa, Italy; Public Health Directorate, Asturias, Oviedo, Spain; Hellenic Health Foundation, Athens, Greece; First Department of Neurology, National and Kapodistrian University of Athens, Athens, Greece; Molecular and Nutritional Epidemiology Unit, Cancer Research and Prevention Institute – ISPO, Florence, Italy; Centre for Cancer Prevention (CPO-Piemonte), Turin, Italy; Human Genetic Foundation (HuGeF), Turin, Italy; Public Health Division of Gipuzkoa, Donostia-San Sebastian, Spain; Department of Epidemiology, German Institute of Human Nutrition, Potsdam, Germany; Inserm, Centre for Research in Epidemiology and Population Health, Institut Gustave-Roussy, Villejuif, France

**Keywords:** Amyotrophic Lateral Sclerosis, Physical activity, Cohort study, EPIC study, Vigorous physical activity, BMI

## Abstract

Previous case–control studies have suggested a possible increased risk of Amyotrophic Lateral Sclerosis (ALS) with physical activity (PA), but this association has never been studied in prospective cohort studies. We therefore assessed the association between PA and risk of death from ALS in the European Prospective Investigation into Cancer and Nutrition. A total of 472,100 individuals were included in the analysis, yielding 219 ALS deaths. At recruitment, information on PA was collected thorough standardised questionnaires. Total PA was expressed by the Cambridge Physical Activity Index (CPAI) and analysed in relation to ALS mortality, using Cox hazard models. Interactions with age, sex, and anthropometric measures were assessed. Total PA was weakly inversely associated with ALS mortality with a borderline statistically significant trend across categories (*p* = 0.042), with those physically active being 33 % less likely to die from ALS compared to those inactive: HR = 0.67 (95 % CI 0.42–1.06). Anthropometric measures, sex, and age did not modify the association with CPAI. The present study shows a slightly decreased—not increased like in case–control studies—risk of dying from ALS in those with high levels of total PA at enrolment. This association does not appear confounded by age, gender, anthropometry, smoking, and education. Ours was the first prospective cohort study on ALS and physical activity.

**Electronic supplementary material:**

The online version of this article (doi:10.1007/s10654-016-0119-9) contains supplementary material, which is available to authorized users.

## Introduction

Amyotrophic Lateral Sclerosis (ALS) is a progressive motor disease characterised by degeneration of the upper and lower motor neurons, with a median survival of 3 years [1]. Cigarette smoking is the only environmental factor which has been shown to increase the risk for this disease in case–control and cohort studies with solid design [2–5].

The observation that ALS incidence in Italy was increased among football players [6], recently reproduced in USA among American Football players [7], is consistent with the hypothesis, among others, that intense physical activity (PA) could be a risk factor for developing ALS. This hypothesis was firstly prompted by a US case–control study showing an increased risk of ALS among people who reported having participated in organised sports in high school [8]. It is supported by another small pilot case–control study from the USA showing an increased risk of ALS among those reporting more leisure-time PA, and those sweating while working [9]; by a more recent large population-based case–control study showing that leisure-time (but not occupational or vigorous) physical activity was associated with ALS (although there was not a clear dose–response relationship) [10]; and by a large population-based case–control study in Japan showing doubled risk of ALS among those practicing vigorous physical activity compared to physically inactive [11]. Conversely, a previous Dutch case–control study did not observe an association between PA and ALS: there was a younger age of symptom onset among those with a higher cumulative leisure PA [12]; although this was potentially explained by a birth cohort effect [13]. A very recent large case–control study found an inverse association between physical activity and ALS [14]. The main epidemiological observations to date on physical activity and ALS are summarised in Table 1.

**Table 1:** Summary of current epidemiological evidence investigating the association between physical activity and Amyotrophic Lateral Sclerosis

References	Setting Study design	Sample	Main association	Confounders
Pupillo et al. [14]	Europe Population-based case–control study	652 ALS cases and 1166 controls	Total PA OR 0.65 (0.48–0.89)	Models adjusted for age, education, BMI, register, interview, traumatic events, coffee, alcohol, smoking, and work-related PA
Yu et al. [15]^a^	Michigan (USA) Case–control study (controls recruited through health-related website)	66 ALS cases and 66 matched controls (sex and age)	High PA intensity in the past 30 years OR 5.98 (0.38–93.3)	
Gotkine et al. [16]^b^	Jerusalem, Israel Comparison of self-reported exposure to Triathlon in a case series with nationwide number of participants in the total population	185 cases of ALS and total Israeli population in 2011 (3,493,700)	OR for participation in triathlon 16.2 (5.6–36.4)	–
Huisman et al. [10]^c^	The Netherlands Population-based case–control study	636 ALS cases and 2166 controls	Total PA adjusted OR 1.02 (0.98–1.06) Leisure time PA OR 1.08 (1.02–1.14) Occupational PA OR 1.00 (0.96–1.04) Vigorous PA OR 1.24 (0.96–1.61)	Models adjusted for gender, age, BMI, current smoking, current alcohol consumption, and level of education
Lehman et al. [7]	USA Standardised mortality ratios among professional American Football players	7 ALS deaths among 3439 American football players	SMR 4.31 (1.73–8.87)	
Vanacore et al. [17]	USA Case–control study on the US mortality dataset	14,628 ALS deaths and 58,512 matched controls (age, gender and geographic area)	OR for intense PA at work 0.95 (99 % CI 0.86–1.04) in men and 1.00 (99 % CI 0.82–1.20) in women	
Chio’ et al. [18]^d^	Italy Standardised mortality ratios among football, basketball player and road cyclist	8 ALS cases among 7325 football players; no ALS cases among 1973 basketball players and 1701 road cyclists	SMR for football players 6.45 (95 % CI 2.78–12.70)	
Okamoto et al. [11]	Japan Population-based case control study	183 ALS cases and 366 matched controls (age and gender)	Vigorous physical activity OR 2.0 (95 % CI 1.0–4.0)	Age, sex, bone fracture, “hate to lose personality”, self-reported stress, type A personality, smoking, alcohol, intake of green/yellow vegetables
Chio’ et al. [6]	Italy Standardised mortality ratios among football players	5 ALS cases among 7325 football players	SMR 6.5 (95 % CI 2.1–15.1)	
Valenti et al. [19]	Italy Population-based case–control study	300 ALS cases and 300 matched controls (age and sex)	Any sport OR 0.38 (95 % CI 0.25–0.58)	
Veldink et al. [12]	Netherlands Case–control study (cases chose their own controls)	219 ALS cases and 254 controls	Cumulative occupational PA OR 4th versus 1st quartile 1.07 (estimated 95 % CI 0.9–4.0) Cumulative leisure PA OR 4th versus 1st quartile 0.8 (estimated 95 % CI 0.6–1.8)	Sex, age, level of education, smoking, alcohol use, and premorbid body mass index (BMI)
Scarmeas et al. [20]	New York (USA) Hospital-based case–control study (controls with other neurological diseases)	431 ALS cases and 152 controls (but analyses based on smaller numbers)	Varsity athlete OR 1.89 (95 % CI 1.05–3.40) Based on 232 cases and 121 controls	Age, sex, always slim, BMI
Longstreth et al. [8]	Washington (USA) Washington state Population-based case–control study	174 ALS cases and 348 matched controls (sex and age)	Lifetime PA in the workplace OR 3rd versus 1st tertile 1.07 (95 % CI 0.57–2.03) Lifetime leisure PA OR 3rd versus 1st tertile 1.46 (95 % CI 0.89–2.39) Participation in organised sports during high school OR 1.52 (95 % CI 1.03–2.25)	Education
Strickland et al. [9]	Minnesota (USA) Semi-population-based semi-hospital-based case–control study	25 ALS cases and 50 controls: 25 hospital-based and 25 population-based	Having received recognition for organised sport participation at school OR 30 (95 % CI 1.04–9.30) How often did PA case you to sweat at work in your twenties OR for trend across 5 categories 1.60 (95 % CI 1.1–2.4) How often did you sweat during leisure time PA in your twenties OR for trend across 5 categories 1.60 (95 % CI 1.1–2.5)	

^a^Single sports (jogging/running, cycling, swimming, aerobic dancing, recreational dancing, calisthenics, gardening, weightlifting, soccer, football, baseball, field hockey, golf, ice hockey, tennis, boxing, wrestling) also tested yielding to non-significant differences between cases and controls

^b^Severe flaws in the study as the study design is not appropriate and prevalence of triathlon in the general population is likely to be overestimated (ever registered with the triathlon association)

^c^None of the variables were associated with ALS survival

^d^Data on football players are an update of the study published on Brain 2005; 128: 472–476

Hypotheses other than that of an association between PA and ALS which are indicated by the evidence produced so far include: (1) that participating in sports (and not PA per se) can increase the risk of ALS (for example via repeated head or limb traumas, or ingestion of illicit substances enhancing sportive performance); or (2) that the age when physical activity is undertaken modifies the risk of ALS. On the other hand, being lean and athletic might be a phenotypic expression of genetic susceptibility to ALS; this might be supported by the finding that cardiovascular fitness was found to be associated with ALS in a record linkage study [21]. To our knowledge, the association between PA and risk of ALS has never been investigated in a prospective study.

In a recent report from the EPIC cohort, a lower body mass index (BMI) in men and a lower waist-to-hip ratio (WHR) in women were associated with an increased risk of dying from ALS [22]. Similar results of a decreased risk in both men and women with increased BMI were recently reported in five US cohorts [23]. To what extent this association is modified by PA has not been explored so far.

The aims of this study are to: (1) assess the association between total PA and risk of death from ALS in the European Prospective Investigation into Cancer and Nutrition (EPIC); (2) explore the contribution of other cumulative measures of PA (occupational, household, recreational, potentially traumatic, practising sports, and vigorous PA) on the risk of ALS; and (3) investigate to what extent these associations are confounded or modified by anthropometric measures or other factors (sex and age).

## Methods

### Participants

The EPIC study was approved by the ethical committee of the International Agency for Research on Cancer (IARC) and by the ethics committees of each participating centre; all participants signed an informed consent. Ninety-one percent of the 518,408 participants were aged 35–70 years and were recruited from the general population residing in defined geographical areas between 1992 and 2002, in 23 centres across 10 Western European countries (Norway, Sweden, Denmark, United Kingdom, Netherlands, Germany, France, Spain, Italy, and Greece) [24]. Exceptions were the French cohort (based on women members of the health insurance for state school), the Ragusa (Italy) cohort (based on blood donors and their spouses), the Utrecht (Netherlands) and Florence (Italy) cohorts (based on breast cancer screening participants), and part of the Oxford (UK) cohort (based on vegetarians and vegans) [24]. The Norway, France, Naples (Italy), and Utrecht cohorts were restricted to women.

At recruitment, information on lifestyle and dietary habits was collected through standardised questionnaires and anthropometric characteristics were measured. Follow-up for mortality and specific causes of death is carried out actively or through linkage with mortality registries at regional and national levels [24]. To date, follow-up is 98.5 % complete. The Norwegian EPIC sub-cohort (n = 37,185) was excluded from the present analysis because it did not give rise to any ALS cases, given its younger age composition.

Information on mortality and causes of death was collected independently: follow-up time was censored in case of dropout, loss of follow-up or fatal events other than ALS death (whichever occurred first). Each EPIC centre had a different last date of follow-up, based on when a drop in the number of reported causes of death was observed, for minimising false negatives [22]. This resulted in censoring follow-up time at some stage between June 2005 and June 2009, generating 5,815,773 person-years for 472,100 subjects after excluding 9123 (1.9 %) individuals with missing data on exposure or confounding variables.

### Case ascertainment

ALS cases were defined as those subjects for whom “motor neuron disease” (G12.2 according to the 10th revision of the International Statistical Classification of Diseases, Injuries and Causes of Death) was reported as an immediate, antecedent or underlying cause of death (for more details, see [2]).

### Physical activity assessment

The assessment of PA measures is described in detail elsewhere [24, 25]. In brief, participants replied to a questionnaire at baseline about occupational PA, and recreational PA, including duration and frequency of walking, cycling, gardening (average values in summer and winter), household work, do-it-yourself (DIY) activities, and sports during the previous year.

Total PA was investigated using the Cambridge Physical Activity Index (CPAI), which combines occupational PA (sedentary occupation, standing occupation, manual/heavy manual work) with time participating in sports and spent cycling [26]. The total hours per week spent cycling or participating in sports was categorised in four levels (no, ≤3.5 h/week; >3.5 and ≤7.0 h/week; >7.0 h/week); based on a 4 × 4 matrix, participants were then classified in four final categories: inactive (sedentary job, no leisure time PA); moderately inactive (standing occupation and no leisure time PA or sedentary occupation and ≤3.5 h/week of leisure time PA); moderately active (manual occupation and no leisure time PA, or standing occupation and ≤3.5 h/week of leisure time PA, or sedentary occupation and >3.5 but ≤7 h/week of leisure time PA): and active (sedentary job with >7 h/week of leisure time PA, or standing job and >3.5 h/week of leisure time PA, or manual job and any leisure time PA, or heavy manual job) [26]. The index was developed by comparing the EPIC PA questionnaire with objective measures of cardio-respiratory fitness and energy expenditure assessed by heart-rate monitoring with individual calibration (measures validated against gold standard techniques) [26], and recently revalidated against a brief questionnaire examining energy expenditure and time spent in moderate and vigorous physical activity [27].

Information on housework, DIY, gardening and climbing stairs was combined to estimate the overall amount of household activity. Recreational PA included walking, cycling, gardening, sports, and DIY. Potentially traumatic PA was defined as performing manual or heavy manual work, playing sport, or doing DIY. Duration and frequency were directly estimated, and intensity (i.e. energy expenditure) was estimated by assigning metabolic equivalents (METs), ranging from 3 for walking and household activities to 6 for sports [28]. Household and recreational PA were categorised in quartiles of distribution; playing sports was categorized as 0, <12 (below the median) or 12+ (above the median) MET-h/week; vigorous PA was categorized as 0, ≤2 (below the median), or >2 (above the median) h/week; potentially traumatic PA was classified as ever/never.

### Statistical analysis

Cox proportional hazard models, with age as the main time variable were used to investigate the associations between type and amount of PA and ALS mortality. Hazard ratio (HR) estimates were derived for the entire sample, stratified by age and centre of recruitment. Potential confounders included sex, highest level of education attained (none/primary, technical, secondary, university, undetermined), a composite smoking variable (never smoker, former smoker ≥10 years prior to enrolment; former smoker <10 years prior to enrolment, current smoker 1–4 cigarettes/day; 5–14 cigarettes/day, 15–24 cigarettes/day, ≥25 cigarettes/day, undetermined), BMI in units (kg/m^2^) and WHR. This composite smoking variable best takes into account the complex pattern of smoking variables on ALS risk found in this cohort [22].

For each Cox regression analysis, a *p* value for linear trend across categories was calculated by introducing the ordinal variable in the model. The *p* value for interaction between each PA category and sex, age (<50 years and 50+ years), BMI, and WHR was estimated using the log-likelihood ratio test comparing models with and without the interaction term (allowance made for *p* < 0.100). To further explore to what extent anthropometric measures could act as confounders separate models were computed: (1) including and excluding them; and (2) stratified by BMI categories [under/normal weight (<25.0 kg/m^2^), overweight (25.0–29.9 kg/m^2^), and obese (≥ 30 kg/m^2^)] and WHR sex-specific quartiles. To explore the extent to which PA modifies the age of ALS onset, the analysis of each PA variable was repeated among those with an age of death from ALS <70 years (N = 120), and among those aged 70+ years (N = 102).

A sensitivity analysis was conducted with models excluding ALS cases arising during the first 3 years of follow-up in order to minimise the potential for reverse causation. Statistical significance was set at 5 % for two-tailed tests.

## Results

The characteristics of the cohort participants by the CPAI are described in Table 2.

**Table 2:** Demographic and lifestyle characteristics of the cohort according to categories of physical activity (n = 472,100)

	Inactive N = 109,545	Moderately inactive N = 161,906	Moderately active N = 113,854	Active N = 86,795
*Gender*				
Men, N (%)^a^	29,501 (26.9)	46,110 (28.5)	36,309 (31.9)	36,315 (41.8)
Women, N (%)^a^	80,044 (73.1)	115,796 (71.5)	77,545 (68.1)	50,480 (58.2)
Age (years), mean (SD)	55.2 (10.6)	51.8 (9.9)	49.8 (10.0)	47.9 (12.2)
*Smoking* ^b,c^				
Never	58,513 (54.2)	83,126 (52.2)	56,715 (50.7)	38,598 (44.9)
Former ≥10 years	17,003 (15.8)	28,405 (17.8)	21,147 (18.9)	17,411 (20.3)
Former <10 years	8535 (7.9)	14,269 (9.0)	10,461 (9.4)	8912 (10.4)
Current 1–4 cig/day	2200 (2.0)	4103 (2.6)	3009 (2.7)	2542 (3.0)
Current 5–14 cig/day	7406 (6.9)	10,953 (6.9)	7671 (6.9)	6748 (7.9)
Current 15–24 cig/day	8661 (8.0)	11,394 (7.2)	7812 (7.0)	7309 (8.5)
Current 25+ cig/day	5607 (5.2)	7086 (4.5)	5108 (4.6)	4457 (5.2)
*BMI*				
Under-/normal weight^b^	44,599 (40.7)	85,665 (52.9)	64,251 (56.4)	46,191 (53.2)
Overweight	41,731 (38.1)	55,710 (34.4)	37,227 (32.7)	31,151 (35.9)
Obese	23,215 (21.2)	20,531 (12.7)	12,376 (10.9)	9453 (10.9)
*Education* ^b,d^				
None/primary	48,254 (46.6)	44,127 (28.3)	28,557 (25.9)	25,043 (29.6)
Technical	17,768 (17.1)	32,871 (21.1)	24,167 (21.9)	24,826 (29.4)
Secondary	18,272 (17.6)	34,953 (22.4)	25,134 (22.8)	14,673 (17.4)
University	19,340 (18.7)	44,210 (28.3)	32,526 (29.5)	19,976 (23.6)
ALS cases	72 (0.07)	80 (0.05)	38 (0.003)	29 (0.03)

^a^Percentages in rows; ^b^ percentages in column; ^c^ smoking status unknown for 6939 individuals (1.5 %); ^d^ unknown for 17,403 individuals (3.7 %)

A total of 219 ALS deaths (76 men and 143 women) arose during follow-up, with the mean follow-up period being 13 years (SD 3 years). Men and younger people were more likely to be physically active; a moderate association between higher levels of PA and higher educational level, lower BMI, and less smoking was observed (Table 2).

**Fig. 1:**
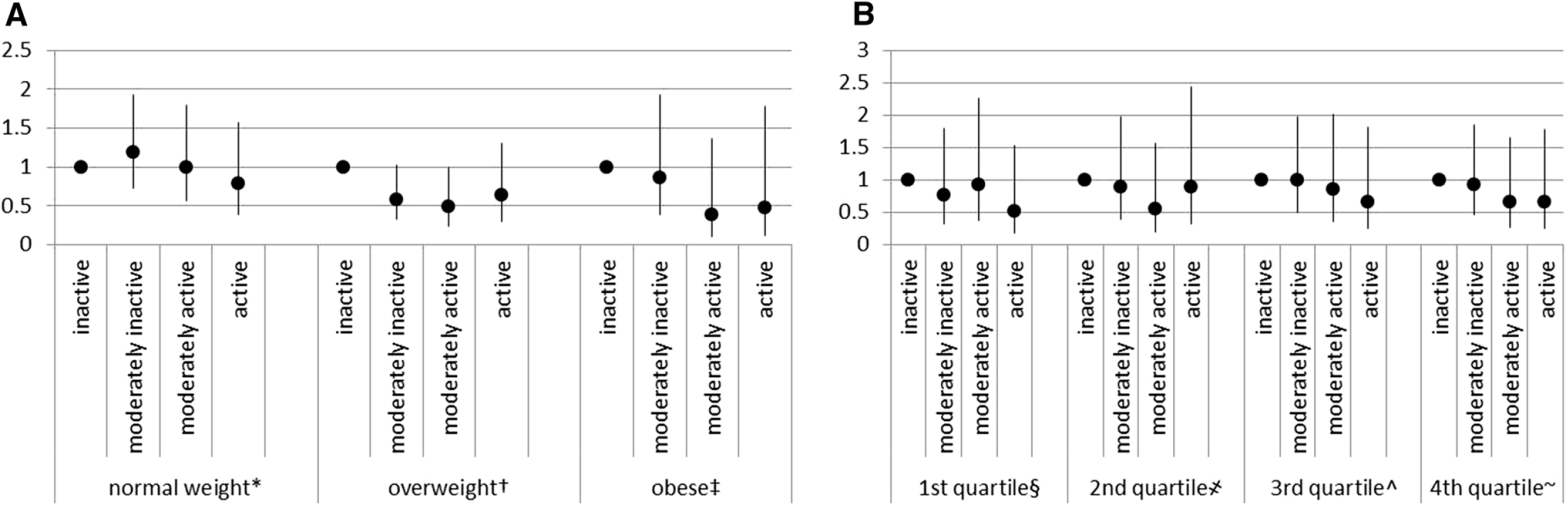
HR and relative 95 % CI for categories of Cambridge Index of physical activity by BMI category (**a**) and quartiles of WHR (**b**). *p value for trend across categories *p* = 0.455; ^†^ *p* value for trend across categories *p* = 0.101; ^‡^ *p* value for trend across categories *p* = 0.119. ^§^ *p* value for trend across categories *p* = 0.340;  *p* value for trend across categories *p* = 0.588; ^^^ *p* value for trend across categories *p* = 0.410; ^~^ *p* value for trend across categories *p* = 0.296

Total PA as estimated by the CPAI was inversely associated with ALS mortality, with a statistically significant trend across categories (*p* = 0.042) in the fully adjusted model (Table 3). Introducing BMI into the model, the statistical significance of the linear trend was maintained (*p* = 0.032), and risk estimates were slightly strengthened (*p* value for the interaction with BMI = 0.318); replacing BMI with WHR, the risk estimates were slightly reduced as was the significance level of the linear trend (*p* = 0.084) (*p* value for the interaction with WHR = 0.889). Hazard ratio estimates by BMI categories and WHR quartiles are shown in Fig. 1.

**Table 3:** HR of dying from ALS according to type of physical activity

	ALS cases	Adjusted HR (95 % CI)^a^	Adjusted HR (95 % CI)^a^ including BMI	Adjusted HR (95 % CI)^a^ including WHR
*Cambridge index of physical activity*				
Inactive	72 (32.9)	1.00 (ref)	1.00 (ref)	1.00 (ref)
Moderately inactive	80 (36.5)	0.88 (0.63–1.22)	0.86 (0.62–1.20)	0.92 (0.64–1.34)
Moderately active	38 (17.4)	0.70 (0.46–1.05)	0.68 (0.45–1.03)	0.76 (0.48–1.20)
Active	29 (13.2)	0.67 (0.42–1.06)	0.65 (0.41–1.04)	0.67 (0.40–1.11)
p-trend		0.042	0.032	0.084
*Occupational activity*				
Sedentary	28 (35.4)	1.00 (ref)	1.00 (ref)	1.00 (ref)
Standing	34 (43.0)	1.39 (0.83–2.35)	1.39 (0.83–2.35)	1.73 (0.97–3.09)
Manual/heavy manual	17 (21.5)	1.28 (0.67–2.44)	1.28 (0.67–2.43)	1.61 (0.80–3.24)
p-trend		0.341	0.343	0.113
*Household activity*				
1st quartile	42 (20.0)	1.00 (ref)	1.00 (ref)	1.00 (ref)
2nd quartile	69 (32.9)	1.43 (0.97–2.11)	1.43 (0.97–2.11)	1.77 (1.11–2.78)
3rd quartile	42 (20.0)	0.82 (0.52–1.27)	0.81 (0.52–1.27)	0.94 (0.57–1.56)
4th quartile	57 (27.1)	1.11 (0.72–1.70)	1.11 (0.73–1.71)	1.25 (0.77–2.02)
p-trend		0.649	0.662	0.795
*Recreational activity*				
1st quartile	46 (21.9)	1.00 (ref)	1.00 (ref)	1.00 (ref)
2nd quartile	56 (26.7)	1.06 (0.71–1.57)	1.05 (0.70–1.55)	1.07 (0.70–1.63)
3rd quartile	59 (28.1)	1.15 (0.77–1.70)	1.13 (0.76–1.67)	0.93 (0.60–1.45)
4th quartile	49 (23.3)	0.91 (0.60–1.39)	0.89 (0.59–1.36)	0.83 (0.53–1.31)
p-trend		0.770	0.688	0.328
*Practising sports*				
No	131 (29.8)	1.00 (ref)	1.00 (ref)	1.00 (ref)
Below the median	36 (16.4)	0.85 (0.58–1.24)	0.84 (0.57–1.23)	0.82 (0.54–1.25)
Above the median	52 (23.7)	1.03 (0.74–1.44)	1.01 (0.72–1.41)	1.01 (0.70–1.45)
p-trend		0.985	0.921	0.899
*Potentially traumatic physical activity*				
Never	90 (41.1)	1.00 (ref)	1.00 (ref)	1.00 (ref)
Ever	129 (58.1)	0.93 (0.69–1.26)	0.92 (0.67–1.24)	0.91 (0.65–1.26)
*Vigorous physical activity*				
None	96 (60.4)	1.00 (ref)	1.00 (ref)	1.00 (ref)
≤2 h/week	39 (24.5)	1.16 (0.77–1.75)	1.15 (0.77–1.74)	1.05 (0.65–1.68)
>2 h/week	24 (15.1)	0.89 (0.55–1.43)	0.89 (0.55–1.43)	0.93 (0.55–1.57)
p-trend		0.789	0.769	0.833

^a^Model stratified by centre and age and adjusted for smoking status, highest level of education attained, and sex

The association between CPAI and ALS mortality did not appear to be modified by gender (*p* interaction = 0.272) or age (*p* interaction = 0.875). When the main analysis was repeated after excluding those who were unemployed at recruitment (34.7 %) (and thus a priori classified as in a sedentary job occupation), the association between CPAI and ALS remained of similar magnitude although falling short of statistical significance due to the reduction of the sample size (ALS cases = 147).

**Fig. 2:**
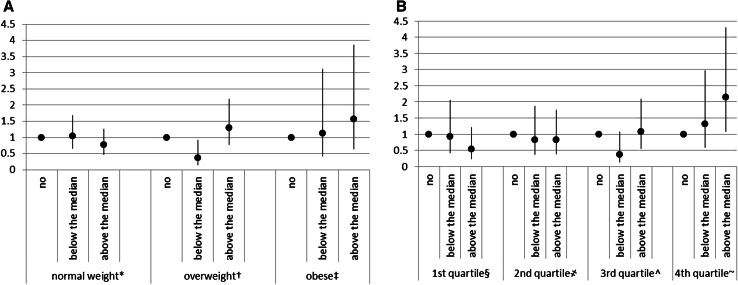
HR and relative 95 % CI for categories of practising sport by BMI categories (**a**) and WHR sex-specific quartiles (**b**). *p value for trend across categories *p* = 0.345; ^†^ *p* value for trend across categories p = 0.633; ^‡^ *p* value for trend across categories *p* = 0.354. ^§^ *p* value for trend across categories *p* = 0.150;  *p* value for trend across categories *p* = 0.577; ^^^ *p* value for trend across categories *p* = 0.878; ^~^ *p* value for trend across categories *p* = 0.033

Occupational, household, recreational, potentially traumatic, sportive, and vigorous PAs were not associated with ALS mortality (Table 3). Although this sample is not powered to allow subgroup analyses, we obtained suggestive results for two interactions. The association with practising sports appeared modified by anthropometry: those normal weight practising sports above the median had a HR 0.77 (95 % CI 0.47–1.26) compared to those who did not practice any sport, those overweight a HR 1.30 (0.77–2.20), and those obese a HR 1.56 (0.63–3.87) (*p* value for interaction with BMI 0.098); subjects in each quartile of WHR had a HR 0.53 (0.23–1.22), HR 0.82 (0.38–1.76), HR 1.08 (0.56–2.09), and HR 2.15 (1.08–4.31) respectively comparing those practising sport above the median versus those not practicing sports (*p* value for interaction with WHR 0.045). These findings were not statistically significant, possible due to small numbers; the association with ALS appears to reverse if sports were practised by obese people (Fig. 2). The association with vigorous PA appeared to be modified by age at recruitment (thus age when the exposure was assessed) (*p* value for interaction = 0.048): despite the reduced sample size (ALS cases = 31), there was a suggestion of a positive association among those reporting vigorous activity early in life (<50 years) HR = 3.20 (95 % CI 0.87–11.74) for ≤2 h/week; and HR = 3.37 (95 % CI 0.88–12.93) for >2 h/week); *p* value for trend across categories 0.070). This association was not present among those reporting vigorous PA later on [HR = 1.04 (95 % CI 0.67–1.62); HR = 0.74 (95 % CI 0.44–1.26), respectively]. Models run separately for early and late age of ALS onset showed effect estimates comparable to those of the main analysis in both categories (results not shown).

The sensitivity analysis involving models excluding ALS cases arising during the first 3 years and during the first 5 years of follow-up showed a stronger inverse association between the CPAI and risk of dying from ALS [compared to the inactive, HR for the moderately inactive = 0.79 (95 % CI 0.56–1.12); HR for moderately active = 0.60 (95 % CI 0.39–0.93); and HR for the active = 0.65 (95 % CI 0.40–1.05), *p* for linear trend = 0.027 after excluding cases arisen during the first 3 years of follow up (N = 197); the same estimates after excluding cases arisen during the first 5 years of follow-up are HR = 0.69 (95 % CI 0.47–1.02); HR = 0.55 (95 % CI 0.34–0.90); and HR = 0.70 (95 % CI 0.42–1.16), *p* for linear trend = 0.067 (n = 162)]. All other results were substantially unchanged, apart from the widening of the confidence intervals due to the reduction of sample size.

## Discussion

The present study shows a borderline significant inverse dose–response relationship between total PA measured at enrolment and subsequent risk of dying from ALS. This association does not appear to be confounded or modified by age, gender, or BMI, smoking, or highest level of education attained; but it became of borderline statistical significance once introducing WHR into model. The CPAI was designed for ranking the participants according to their overall PA based on heart rate monitoring, best reflecting the amount of energy spent in physical activities [26].

This is the first prospective cohort study assessing the type and amount of PA before disease onset, and the subsequent risk of developing and dying from ALS. The finding of an inverse association between PA and risk of ALS is at odds with the majority of findings coming from case–control studies [8, 10, 12, 20].

To date, in fact, all published evidence on the association between ALS and PA comes either from case–control studies [8, 9, 20, 29], or from retrospective cohort studies [6, 7]. The former have the limitation of being prone to recall bias (which might be particularly relevant when assessing PA whose quantification is not easy, and which can be easily related by patients to a disease impairing movements). The retrospective cohort studies investigating the risk of ALS among football players have the advantage of including large numbers, but are limited to a highly selected group, and cannot fully account for potential confounders.

Possible ways in which PA may increase the risk of ALS involve oxidative stress, glutamate excitotoxicity and complex interactions with ALS susceptibility genes [30]. On the other hand, the mechanisms through which PA might protect against ALS are thought to be mediated by exercise-induced changes in motor neuron morphology, muscle-nerve interaction, glial activation, and altering levels of gene expression of anti-apoptotic proteins and neurotrophic factors [31]. The effects of exercise upon astrocytes and angiogenesis are also relevant in ALS as astrocytes play an important structural role in the maintenance of the blood–brain barrier (BBB) [32]. A disruption of the BBB in SOD1 mice was found starting in the early stages of the disease [33]. The extensive disruption of the neurovascular unit may promote the progressive loss of motor neurons, with their integrity dependent upon efficient capillary influx of nutrients and efflux of waste, as well as direct supportive interactions with astrocytes. In the brains of exercised rats, the neurovascular unit is strengthened by increased angiogenesis and astrocyte proliferation [34].

The present data suggest that reverse causality is not a likely explanation of the results: after removing ALS deaths during the first 3 years of follow up, the inverse association strengthened (albeit with a reduced sample size). The role of unemployment in contributing to the CPAI is more complex: it might represent an effect of residual confounding of socio-economic status, or an unmeasured reverse causality. However, the fact that an association of similar magnitude between CPAI and ALS mortality (albeit not significant) is obtained after excluding those subjects who were unemployed at recruitment suggests that residual confounding or reverse causality are perhaps not the most likely explanations.

The present study had lower statistical power to detect interactions, and the findings in this sense should be interpreted cautiously and regarded as hypothesis generating. Nevertheless, it is interesting that there was also a suggestion of an increased risk of ALS from vigorous PA in obese subjects (or subjects with an elevated BMI due to substantial increase of muscle mass) among those practising sports, and in young individuals. Should these be confirmed by further investigations, they may reflect some specific characteristics of these two categories, such as, for example, the use of some stimulating agents enhancing sportive performance and/or promoting weight loss [35]. Some of these compounds, such as anabolic steroids and testosterone, increasingly used and abused by athletes for enhancing performance [36], have been shown to stimulate muscle mass increase [37]. These hormones may increase ALS risk though unknown mechanisms, and may also contribute to the higher incidence of ALS in men than women [38]. This hypothesis is consistent with previous observations linking ALS risk to practising sports in general [17, 20], or being professional football players [6, 7], or varsity athletes [8, 9, 20] or professional athletes [17]. Also, a recent report from Europe describes an inverse association between PA and ALS, except for professional sport players [29, 39]. Unfortunately, we are not able to reproduce the findings on professional sport players in this setting, as this information is not available in EPIC. A cohort effect explaining the suggested increased risk among those practising vigorous PA at younger ages has already been suggested by Ascherio [13] in reference to the study by Veldink et al. [12]; this cannot be ruled out, although it seems an unlikely explanation given that it applies to vigorous activity only. The small sample size and the relative oversampling of women by design in the EPIC study do not allow powered estimates of association in both sexes, separately. Despite the *p* value for interaction with sex being far non-significant (*p* = 0.272), we cannot rule out that the present results are at least partially driven by the association in women.

Death records appear to be reliable for ascertaining ALS deaths (and hence ALS itself) in this [2] and other large population studies [40]. Nonetheless, a recent study in UK identified an error in the WHO ICD-10 Alphabetic coding Index where progressive supranuclear palsy was mistakenly given as code G12 [23]. However, a validation exercise on death records demonstrated that only 8 % of the women receiving a diagnosis of ALS were misclassified [3]. Furthermore, misclassification introduced by the use of death records instead of specialist diagnoses would likely be non-differential and thus bias the estimate towards the null; therefore the risk estimates reported here might underestimate true associations.

In summary, in this prospective study, practising more PA was associated with a reduced risk of dying from ALS. This association does not appear to be confounded or modified by age, gender, BMI, smoking and the highest level of education attained, and it is unlikely explained by reverse causation.

## Electronic supplementary material

Below is the link to the electronic supplementary material.

Supplementary Fig. 1 Matrix for the adjudication of the Cambridge Index of Physical Activity categories starting from type of job and duration of sport and cycling in hors/week in the EPIC study (JPEG 78 kb)

Supplementary material 2 (DOCX 15 kb)
