# Supplementary figures and images for: Physical activity and risk of Amyotrophic Lateral Sclerosis in a prospective cohort study
Source: Eur J Epidemiol. 2016 Mar 11;31:255–66. doi: 10.1007/s10654-016-0119-9 (PMC4820490; doi:10.1007/s10654-016-0119-9)

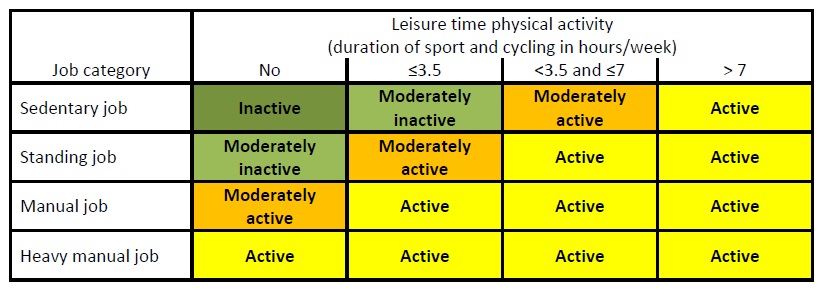

Supplement: Supplementary file 1 — Matrix for the adjudication of the Cambridge Index of Physical Activity categories starting from type of job and duration of sport and cycling in hors/week in the EPIC study (JPEG 78 kb) [file 10654_2016_119_MOESM1_ESM.jpg]
