# Supplementary material for: Physical activity and risk of Amyotrophic Lateral Sclerosis in a prospective cohort study
Source: Eur J Epidemiol. 2016 Mar 11;31:255–66. doi: 10.1007/s10654-016-0119-9 (PMC4820490; doi:10.1007/s10654-016-0119-9)
Supplement: Supplementary file 2 — Supplementary material 2 (DOCX 15 kb) [file 10654_2016_119_MOESM2_ESM.docx]

Supplementary Table 1: Description of the National EPIC cohorts in terms of demographic characteristics, ALS cases, and physical activity measured with the Cambridge Index of Physical Activity

|  |  |  |  |  |  | **Cambridge Index of Physical Activity** | | | |
| --- | --- | --- | --- | --- | --- | --- | --- | --- | --- |
|  | **Cohort size** | **Person-years** | **Women (%)** | **Mean age (SD)** | **ALS cases (%)** | **Inactive (%)** | **Moderately inactive (%)** | **Moderately active (%)** | **Active (%)** |
| Sweden | 53,692 | 666,287 | 30,277 (56.4) | 52.3 (10.9) | 36 (0.07) | 11,468 (21.4) | 19,230 (35.8) | 13,855 (24.3) | 9,139 (17.0) |
| Denmark | 56,962 | 595,277 | 29,838 (52.4) | 56.7 (4.4) | 28 (0.05) | 6,364 (11.2) | 17,368 (30.5) | 13,818 (24.3) | 19,412 (34.1) |
| UK | 84,812 | 973,725 | 59,063 (69.6) | 50.2 (14.4) | 49 (0.06) | 23,212 (27.4) | 28,712 (33.9) | 18,907 (22.3) | 13,981 (16.5) |
| Netherlands | 33,843 | 432,526 | 26,141 (77.2) | 50.1 (11.8) | 20 (0.06) | 2,838 (8.4) | 8,725 (25.8) | 8,834 (26.1) | 13,466 (39.7) |
| Germany | 53,075 | 582,698 | 30,246 (57.0) | 50.7 (8.6) | 14 (0.03) | 9,099 (17.1) | 19,415 (36.6) | 13,854 (26.1) | 10,707 (20.2) |
| France | 74,466 | 1,103,512 | 74,466 (100) | 52.9 (6.7) | 39 (0.05) | 14,462 (19.4) | 29,842 (40.1) | 23,645 (31.8) | 6,517 (8.8) |
| Spain | 41,438 | 538,514 | 25,808 (62.3) | 49.3 (8.0) | 18 (0.04) | 15,926 (38.4) | 13,774 (33.2) | 7,267 (17.5) | 4,471 (10.8) |
| Italy | 46,653 | 546,971 | 32,045 (68.7) | 50.6 (7.9) | 12 (0.03) | 13,737 (29.5) | 17,733 (38.0) | 8,210 (17.6) | 6,973 (15.0) |
| Greece | 27,159 | 261,990 | 15,981 (58.8) | 53.2 (12.6) | 3 (0.01) | 12,439 (45.8) | 7,107 (26.2) | 5,464 (20.1) | 2,149 (7.9) |
